# Neurobehavioral pathways linking socioeconomic status hardship to suicide risk versus resilience in young adolescents: the roles of sleep health and default mode network connectivity

**DOI:** 10.1038/s41398-025-03710-y

**Published:** 2025-11-24

**Authors:** Assaf Oshri, Cullin J. Howard, Steven M. Kogan, Linhao Zhang, Charles F. Geier, Brian W. Bauer, Ellen House

**Affiliations:** 1 Department of Human Development and Family Science, University of Georgia, Athens, GA 30602 USA; 2 Georgia Center for Developmental Science, University of Georgia, Athens, GA 30602 USA; 3 Department of Psychology and Human Development, Vanderbilt University, Peabody College, Nashville, TN 37203 USA; 4 Department of Psychology,, University of Georgia, Athens, GA USA; 5 Department of Psychiatry and Health Behavior, Augusta University/University of Georgia Medical Partnership, Athens, GA 30602 USA

**Keywords:** Depression, Human behaviour

## Abstract

Socioeconomic hardship (SES-H) is a known risk factor for adolescent suicidal thoughts and behaviors (STB). This study examined sleep health as a pathway linking SES-H to suicide risk and evaluated the moderating role of Default Mode Network (DMN) coherence. Data came from three waves of the Adolescent Brain Cognitive Development (ABCD) Study (N = 11,878). Increased SES-H predicted greater suicidal ideation and attempts. Shorter sleep duration mediated the association with ideation, while high DMN coherence attenuated the indirect effect of SES-H on ideation via sleep. Findings highlight sleep health as a mechanism connecting socioeconomic adversity to suicidality and suggest DMN coherence may function as a neuroprotective factor for youth resilience.

## Introduction

Suicide is the second leading cause of death among US adolescents and young adults [1]. Recent data indicates that among high school students, 14% of boys and 27% of girls had seriously considered suicide in the past year [2]. Of particular concern, precursors of suicidality, including suicidal thoughts and behaviors (STBs), are increasingly prevalent among children under age 13 [3]. A recent meta-analysis detected concerning prevalence rates for lifetime suicidal thoughts (15.1%), suicidal attempts (2.6%), and non-suicidal self-injury (6.2%) among pre-adolescents in community samples [4].

STBs are disproportionately prevalent among youth who reside in communities with high concentrations of poverty [5–7]. Low-resource environments promote a range of contextual risks linked to suicidality, including family poverty [8], attendance at resource-poor schools [9] and exposure to community violence [10]. The link between socioeconomic hardship (SES-H) and suicidality is robust, emerging consistently in high, middle, and low-income countries [11]. Less is known, however, regarding (a) the bioregulatory processes linking SES-H to suicidal ideation, and (b) neurobehavioral processes linked to individual differences in youths’ adaptation to SES-H. Sleep quality is critical to adolescent health, yet remains an understudied pathway linking SES-H and STB. Youth from socioeconomically disadvantaged families often experience inconsistent sleep schedules, shorter sleep duration, and greater sleep difficulties, all of which may heighten their risk for suicidal outcomes [12]. Further, Default Mode Network (DMN) coherence, operationalized as within-network functional connectivity at rest, is a promising candidate for understanding individual differences in general, and resilience in particular, in the influence of SES-H on sleep disturbance.

### SES-H, sleep health, and suicide vulnerability among children

Considerable research documents the impact of SES-H on suicidality among adults [13]. Studies consistently show a strong association between unemployment, financial hardship and suicide risk [14]. Events like job loss, eviction, foreclosure, or bankruptcy create immediate financial strain and disrupt a person’s sense of security and identity, leading to feelings of shame, despair, and hopelessness [15, 16]. Adults facing chronic economic hardship often experience social isolation, lack of access to mental health services, and elevated levels of psychological distress [17]. However, prior research indicates that socioeconomic hardship is not limited to acute financial strain, but also reflects an accumulation of stressors across multiple domains [18, 19]. In addition to income-related deprivation, psychosocial stressors may include structural and relational domains of hardship. Stressors such as lower caregiver education can diminish parenting resources and opportunity structures [20, 21], while single-parent household status may strain available time for relational support, parenting, and stability [22]. These domains represent proximal influences that broadly reflect the ecological burden of SES-H that shapes the developmental environment and may have consequences for overall mental health.

Far less research has considered how SES-H affects suicidal vulnerabilities among children and adolescents. For children and adolescents, exposure to SES-H not only precipitates emotional distress but also constitutes a rearing context affecting the development of mental health and suicide-specific vulnerabilities. SES-H increases the probability of children’s exposure to a range of chronic and acute stressors during sensitive developmental periods [23, 24]. Such rearing environments deny children material, social, and intellectual resources and increase the occurrence of acute stressors. Further, lower-SES households may face greater caregiving demands, nonstandard work hours, and daily stressors that make it more difficult to maintain consistent routines for children, particularly around bedtime, which can contribute to sleep problems and shorter sleep durations among youth [25]. These strained rearing environments confer heightened developmental risk by exposing children to unpredictable and inconsistent opportunities to develop adaptive skills and strategies needed to navigate daily challenges [26]. Although a growing number of empirical studies have begun to explore the mechanisms linking SES-H to STBs in children and adolescents [6, 27], additional research is needed to deepen understanding in this area.

Emerging research suggests that sleep health may be a pivotal bioregulatory process in linking socioeconomic adversity to suicidality in children and adolescents [28–31]. Sleep problems are like a bellwether of poor bioregulatory integration and a causal influence undermining effective neuroregulatory systems integration and cognitive processing of stressful events [32, 33]. Sufficient and high-quality sleep regulates and restores the body’s internal environment, supporting efficient metabolic, immune, and brain functioning [32]. Sleep reduces metabolic rate and energy consumption, helping the body conserve resources for critical functions like growth and repair [34]. During sleep, the brain undergoes crucial maintenance, such as memory consolidation and toxin clearance [35]. Resource-poor conditions make sleeping difficult for children *directly* due to proximal factors such as noise, lack of consistency, and anxiety [36]. Growing up with socioeconomic adversity, however, further undermines sleep health as a child’s bioregulatory systems “calibrate” to adapt to the environment in ways that make sleep difficult [37, 38]. Specifically, chronic adversity fosters alterations in stress responsivity associated with sleep duration and quality [38].

Empirical evidence increasingly links disruptions in bioregulatory systems, particularly sleep, to the emotion regulation difficulties and maladaptive cognitive processes associated with STBs [29, 36, 39]. Insufficient sleep can impair memory, attention, and problem-solving [40]. A range of sleep problems, characterized as longer latency, reluctance to go to bed, difficulty falling asleep, anxiety when falling asleep, night awakenings, and difficulty falling asleep after awakenings, as well as chronic sleep deprivation, are linked to increased rates of anxiety, depression, irritability, and behavioral problems [41–43]. Recent studies further connect sleep problems directly to suicidal ideation, planning, and attempts [30, 44–48]. Yet, even beyond these multifaceted sleep problems, a meta-analytic review by Chiu and colleauges [49] identified sleep duration, in particular, as having a dose-response relation to adolescent suicide risk, with longer sleep duration significantly reducing the odds of suicidal ideation and attempts. Given that sleep is a modifiable factor, it is critical for future prospective research to investigate sleep health as a potential mechanism linking SES-H to suicidality in children and adolescents. Improving sleep has been shown to increase emotional and stress regulation [50] which are closely related to suicidal risks. Therefore, the present analysis will consider both overall sleep problems and sleep duration specifically as potential mediators linking SES-H to the development of adolescent STBs.

### Individual differences in adaptation to SES-H: the default mode network

We expect that during the transition to adolescence, exposure to SES-H will forecast STBs via disruptions in sleep health. We acknowledge, however, that considerable individual differences exist in children’s responses to adversity. Work by our team [51–53] and others [54–57] suggests that connectivity in specific resting-state brain networks are linked to children’s ability to adapt to and cope with environmental hardships [58, 59]. This study focuses on the resting state within functional connectivity of the Default Mode Network (DMN). The DMN is a group of interconnected brain regions, including the medial prefrontal cortex, posterior cingulate cortex, precuneus, and angular gyrus, which show increased connectivity when the brain is not actively engaged in a cognitive task, i.e., during ‘rest’ [60]. The DMN supports a range of functions, including introspection, self-referential thought, future planning, and aspects of social cognition, with higher coherence generally linked to efficient intra-network neural communication [61–63].

Neurodevelopmental research indicates that the strength of connectivity among the regions comprising the DMN (also seen as *coherence*) changes significantly throughout childhood and adolescence [64]. In particular, in parallel to brain maturational processes, within-DMN connectivity generally increases from childhood to early adolescence. These neurodevelopmental changes are followed by a decline in coherence from middle adolescence to adulthood as the network becomes more specialized and segregated from other networks [64, 65]. Although studies of older or clinical populations often link heightened DMN coherence to depression, rumination, and other internalizing symptoms [66], research with childhood and early adolescent samples report increased default network connectivity to be linked to positive neurocognitive processes such as enhanced self-referential/introspective processing [67], positive future planning [68], decreased psychopathology [69], and cognitive maturation broadly [65]. From a neurocognitive developmental perspective, increased DMN coherence during earlier stages of development is viewed as a normative process that supports growing integration and efficiency within and across neural systems [64, 65].

The idea that brain circuity serves as an important context for learning is gaining support in cognitive neuroscience [53, 70]. Higher DMN coherence may serve as a neurocognitive context during early adolescence, reflecting age-appropriate neural maturation that may support youth resilience [53]. Recent studies suggest that intra-network connectivity during resting state in the DMN may influence overall socioemotional health in the aftermath of stressful experiences [71–73]. High DMN coherence may enable children to process and reflect on challenging events in a manner that fosters well-being [74, 75]. DMN coherence may foster more “effective” coping by supporting self-referential and future-oriented thoughts that allow a child to organize information and plan responses to stressful environmental conditions. In contrast, relatively low DMN coherence potentially leads to more fragmented or inconsistent information processing [76]. We therefore consider the potential of DMN coherence as a brain context that can modulate (a) the extent to which SES affects sleep duration and quality.

Concerning sleep outcomes, DMN coherence is expected to attenuate the influence of adversity on sleep duration and quality [77, 78]. The DMN supports cognitive reappraisal and emotional self-soothing, helping individuals reinterpret stressors in less threatening ways, potentially reducing stress-related arousal that disrupts sleep [51, 79]. Furthermore, DMN coherence facilitates communication between regions regulating physiological responses to stress, such as the hypothalamus and limbic system, promoting relaxation and a smoother transition to restorative sleep [80, 81]. Indeed, emerging evidence has linked DMN coherence to sleep health, potentially by promoting emotion regulation and reducing attention problems and cognitive arousal near bedtime [77, 82, 83]. We thus hypothesize that SES-H during late childhood will increase sleep problems in early adolescence, primarily among youth with low levels of DMN coherence.

### The current study

Although SES-H is a well-established risk factor for suicidality in children and adults, little research investigates how SES-H increases suicidal vulnerability via bioregulatory systems. SES-H comprises an environment that disrupts bioregulatory integration, particularly impairing sleep quality in developing children, which may increase their risk of suicidal ideation during childhood and pre-adolescence. The extent to which SES-H undermines sleep may depend on global neural processing systems that facilitate or hinder children’s adaptation to challenging rearing environments. We tested the hypotheses regarding the pathways linking SES-H to early adolescents’ STBs. Specifically, we expect that increased exposure to SES-H assessed at age 10 will forecast suicidal ideation and attempts over the next two years. We anticipate that increases in overall sleep problems will mediate this effect and decreases in sleep duration during the intermediary year. We further expect that the influence of SES-H on sleep will emerge primarily in children who have lower levels of DMN coherence.

## Methods

### Sample

We tested the hypotheses with data from the ABCD study of 11,878 youth recruited from 21 sites across the United States, ensuring a diverse representation of socioeconomic, ethnic, and biobehavioral health backgrounds (Data Release 5.0). The study procedures were approved by human research protection programs and institutional review boards at the participating universities (full ABCD study details found at [84, 85]). Both primary caregivers and youth provided informed consent and assent to participate. We analyzed data from 3 time points: baseline (Time 1[T1]; 47.8% female; M_age_ = 9.94; SD = 0.63), T3 (12 months; M_age_ = 10.95; SD = 0.65), and T5 (24 months; M_age_ = 12.05, SD = 0.67). A visualization of the data collection timing for the primary variables used in this analysis is found in supplementary materials (Figure S1). The sample’s racial-ethnic composition was 52.0% European American, 15.0% African American, 20.3% Latino(a), 2.1% Asian/Pacific Islander, and 10.5% Other. The exclusion criteria for the ABCD study included MRI contraindications (e.g., metal implants), lack of English or Spanish fluency, a history of major neurological disorders, premature birth (i.e., under 28 weeks), and hospitalization at birth for more than 30 days [84, 85]. Site-designated clinicians implemented risk assessment procedures with children who reported recent suicidal ideation or attempts.

Neuroimaging data were collected on Siemens, General Electric, or Philips 3 T scanners with 32-channel head coils. The complete neuroimaging protocol for the ABCD study has been published elsewhere [84]. Data for this investigation comes from the resting-state fMRI brain scan that included a continuous 20-minute acquisition of stimuli-free neural functional activity. Although techniques were used to mitigate movement in the scanner (e.g., real-time motion correction and monitoring), the participants’ young age and the scan length resulted in some scan data failing to meet acceptable quality control criteria outlined by Hagler et al. [86]. The current analysis used data from participants who were recommended for inclusion based on MRI quality assessment at T1, were free from MRI incidental findings requiring a clinical follow-up [87], and who completed the suicidal risk assessments at all time points (T1, T3 and T5), resulting in a final sample of 8061 youths.

### Measures

#### SES-H

SES-H at T1 was assessed using several key indicators: caregivers’ marital status (coded 1 = No, 0 = Yes), caregivers’ employment status [1 = Not employed, 0 = Employed (full-/part-time)], family material deprivation, family income-to-poverty ratio, and the neighborhood area deprivation index. Family material deprivation was assessed using seven items from the Parent Demographics Survey, which identified whether a family experienced any financial hardships (e.g., inability to afford food, missed rent or mortgage payments) in the past 12 months, with responses coded as 1 = Yes and 0 = No. The family income-to-poverty ratio was determined by dividing the family’s reported annual income by the federal poverty threshold for the interview year, adjusted for household size. The area deprivation index was a composite measure (converted to national percentiles) based on 17 neighborhood factors (e.g., income, education, employment, housing quality) derived from the American Community Survey (ACS) [88]. For more information on the modeling approach and a more detailed explanation of indicator selection criteria see supplemental material (Table S1).

#### Suicidal ideation and attempts

Children completed a computerized version of the Kiddie Schedule for Affective Disorders and Schizophrenia (K-SADS), which uses the Diagnostic and Statistical Manual of Mental Disorders (Fifth Edition) criteria to assess mental health disorders [89]. At T1, T3, and T5, children responded to questions about *present* (past two weeks) and *past* (lifetime) suicidal ideation and attempts (see Supplemental Table S2 for item-specific details). Two dichotomous outcome variables were created for analysis: (1) T3/T5 suicidal ideation and (2) T3/T5 suicide attempts. Participants reporting any present or past passive, nonspecific active, or active suicidal ideation at T3 or T5 were coded as 1 for T3/T5 suicidal ideation; those reporting none were coded as 0. Similarly, participants reporting any present or past suicide attempts at T3 or T5 were coded as 1 for T3/T5 suicide attempts, with 0 indicating no attempts. T1 suicidal ideation and attempts were coded similarly and included as covariates in all analyses to assess change in suicide risk (i.e., indicated by ΔSuicidal Ideations and ΔSuicide Attempts) beyond baseline levels.

#### DMN coherence

Resting-state scans at T1 were preprocessed by the ABCD Data Analysis and Informatics Core using the standardized ABCD pipeline [86]. Mean resting state functional connectivity (rsFC) was calculated using the Gordon parcellation scheme [90] for 12 predefined resting-state networks, including the DMN. DMN coherence was calculated as the average Fischer r-to-Z correlations for each pairwise combination of regions of interest that belong to the DMN network [86]. A higher rsFC score indicates a higher average Pearson correlation over all pairs of regions within the DMN.

#### Sleep problems and duration

Parents reported on their children’s sleep problems and duration with the Parent Sleep Disturbance Scale for Children at T1 and T3. Sleep problems included issues such as shorter duration, longer latency, reluctance to go to bed, difficulty falling asleep, anxiety when falling asleep, night awakenings, and difficulty falling asleep after awakenings. Items were summed at (T1 and T3 α = 0.73). For sleep duration at T1 and T3, parents answered the question, “How many hours of sleep does your child get on most nights in the past six months?” using a 5-point Likert scale (1 = 9 to 11 h; 2 = 8 to 9 h; 3 = 7 to 8 h; 4 = 5 to 7 h; 5 = less than 5 h). To characterize change in these sleep indices, we computed residualized change scores by regressing each participant’s T3 sleep score on their T1 and saving each participant’s residual [91]. Residualized change scores (i.e., ΔPoor Sleep Duration, and ΔSleep Problems) were used in mediation path analyses, while T1 and T3 scores were employed in bivariate correlations. T1 Sleep Duration and Sleep Problems scores were retained as model covariates to control for baseline levels of these variables.

#### Covariates

All models included baseline child age and biological sex as demographic covariates. Baseline scores on sleep indices (i.e., sleep problems and sleep duration) and suicide risk measures (suicidal ideations and suicide attempts) were also included as covariates on all paths. Additionally, we controlled for child baseline levels of anxiety/depressive symptoms using age- and sex-adjusted anxious/depressed subscale T-scores from the parent-reported Child Behavior Checklist (CBCL) [92].

### Statistical analysis

Hypotheses were tested using a structural equation modeling (SEM) framework in Mplus version 8.3 [93]. A latent SES-H variable at baseline was estimated using confirmatory factor analysis (CFA). Then two separate mediation models were fit to examine sleep duration and sleep problems as mediators in the association between SES-H and youth suicide risk. Unconditional indirect effects from mediation models were estimated using the bootstrapping approach (draws = 5000), providing bias-corrected confidence intervals and robust standard errors for mediation effects even under conditions of non-normality in complex models with categorical/dichotomous outcomes [94]. Last, we tested if SES-H interacted with youth rsFC DMN to predict change in sleep duration/problems and its subsequent effect on suicidal ideations/attempts (i.e., the conditional indirect effect). Conditional indirect effects were tested using the approach outlined by Preacher et al. [95], with regions of significance (RoS) for interactions identified using the Johnson-Neyman approach and the overall conditional indirect effect visualized using pick-a-point simple slope plot [96, 97].

The percentage of missing data on all study variables ranged from 0.00 ~ 22.3%. Because suicide risk indicators were dichotomized, we tested hypotheses using probit SEM models with the weighted least squares with mean and variance adjusted (WLSMV) estimator [98]. Criteria for evaluating fit across models was: ≤ 0.08 for the root mean square error of approximation (RMSEA) and the standardized root mean squared (SRMR), and ≥ 0.90 for comparative fit and Tucker-Lewis indices (CFI/TLI) [99]. We accounted for the multi-site and family-nested nature of the ABCD data set by stratifying by MRI scanner serial number and clustering by family [100]. All statistical tests were two-sided with a significance threshold of *p* < 0.05 (unadjusted), and 95% confidence intervals are reported throughout to reflect the precision of estimates. The code for this statistical analysis is available at https://github.com/GeorgiaCenterforDevelopmentalScience (GCDS@UGA).

## Results

**Fig. 1:**
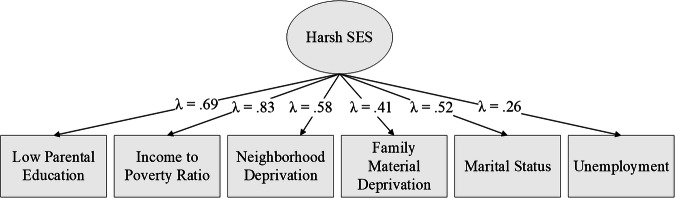
Standardized Factor Loadings from the SES Hardship CFA. Note. All loadings (indicated by λ_ij_) are significant, *p* < 0.001.

Using CFA, we fit a latent factor of SES-H that included low parental education, income-to-poverty ratio, neighborhood and family deprivation scores, parent employment, and marital status at baseline (Fig. 1). The measurement model for the SES-H factor fit the data well (χ2[9] = 210.90, CFI/TLI = 0.959/0.931, RMSEA = 0.05, SRMR = 0.05), and all factor loadings were significant and above 0.25 (*p* < 0.001). A more detailed breakdown of the specific ABCD variables used as SES-H indicators can be found in the supplementary material (Table S1). Participant SES-H latent factor scores were extracted and centered to support usage in subsequent models.

Bivariate correlations and descriptive statistics of study variables are presented in Table 1. Across the study period, 1366 youths reported suicidal ideation, 703 of these cases were unique to T3/T5. Additionally, 218 youths reported attempting suicide over the study period, with 137 of these cases being unique to T3/T5. Considering possible covariates, youth age was associated with sleep duration, as well as suicidal ideations and attempts. Males exhibited significantly more T3 sleep problems (t(8041) = 2.12, *p* < 0.05) and suicidal ideations (χ^2^ [1, 8061] = 7.81, *p* < 0.01) than females. Accordingly, youth age, and biological sex were retained as covariates.

**Table 1:** Bivariate Correlations Among Study Variables.

Variable	1	2	3	4	5	6	7	8	9	10	11
1. SES-H	1										
2. Poor Sleep Duration T1	0.36***	1									
3. Poor Sleep Duration T3	0.33***	0.58***	1								
4. Sleep Problems T1	0.11***	0.44***	0.31***	1							
5. Sleep Problems T3	0.08***	0.27***	0.44***	0.66***	1						
6. Suicidal Ideations T1	0.03**	0.04***	0.03**	0.08***	0.08***	1					
7. ΔSuicidal Ideations T3-5	0.05***	0.06***	0.06***	0.10***	0.12***	0.29***	1				
8. Suicide Attempt T1	0.05***	0.05***	0.04***	0.05***	0.05***	0.31***	0.15***	1			
9. ΔSuicide Attempt T3-5	0.07***	0.05***	0.06***	0.06***	0.06***	0.20***	0.38***	0.29***	1		
10. rsFC DMN	0.00	−0.03*	−0.01	−0.02	−0.03*	−0.01	−0.01	0.00	−0.01	1	
11. Youth Age	−0.03*	0.05***	0.08***	0.02	0.02	0.01	0.03**	0.01	0.03**	0.00	1
M (SD) / 0:1	−0.15 (0.73)	1.67 (0.79)	1.82 (0.84)	11.63 (3.67)	11.85 (3.74)	7398: 663	7068:993	7977:81	7887:172	0.00 (0.07)	9.50 (0.51)

***p < 0.001, **p < 0.01, *p <0.05.

### Mediation models

SEM estimates for the sleep problems mediation model are presented in Table 2a. SES-H was directly linked to an increase in the probability of youth reporting later suicidal ideations (β = 0.06, 95% CI [0.02, 0.09], *p* < 0.01) and suicide attempts (β = 0.18, 95% CI [0.11, 0.24], *p* < 0.001). Although T3 sleep problems were associated with an increased probability of suicidal ideations (β = 0.08, 95% CI [0.05, 0.12], *p* < 0.01), the association between SES-H and sleep problems was nonsignificant (β = 0.01, 95% CI [−0.01, 0.04], *p* = 0.31). As a result, the indirect effects estimated for SES-H to suicidal ideations and attempts through changes in sleep problems at T3 were both nonsignificant.

**Table 2:** **a**. Summary of the Mediation Model Predicting Suicidal Ideations and Attempts via Sleep Problems. **b**. Summary of the Mediation Model Predicting Suicidal Ideations and Attempts via Poor Sleep Duration.

a									
	ΔSleep Problems			ΔSuicidal Ideations (probit)			ΔSuicide Attempts (probit)		
Variables	b	β	95% CI[LB, UB]	b	β	95% CI[LB, UB]	b	β	95% CI[LB, UB]
**Unconditional Mediation**									
SES Hardship	0.01	0.01	[−0.01, 0.01]	**0.32**	**0.06**	[0.13, 0.51]**	**1.01**	**0.18**	[0.66, 1.38]***
T1 Anxious-Depressed	**0.07**	**0.09**	[0.04, 0.09]***	**1.14**	**0.14**	[0.87, 1.40]***	1.14	**0.13**	[0.69, 1.49]***
ΔSleep Problems				**0.97**	**0.08**	[0.57, 1.36]***	0.31	0.03	[−0.49, 1.02]
R^2^		0.009**			0.139***			0.157	
**Indirect Effects**									
SES Hardship → ΔPoor Sleep Duration → Suicide Risk									
			*Direct*	**0.32**	**0.06**	[0.13, 0.51]**	**1.01**	**0.18**	[0.66, 1.38]***
			*Indirect*	0.01	0.00	[−0.01, 0.02]	0.00	0.00	[−0.00, 0.01]
**Conditional Mediation**									
SES Hardship	0.01	0.01	[−0.01, 0.02]	**0.31**	**0.05**	[0.12, 0.50]**	**0.99**	**0.17**	[0.64, 1.36]***
T1 Anxious-Depressed	**0.07**	**0.09**	[0.04, 0.09]***	**1.14**	**0.14**	[0.87, 1.40]***	**1.09**	**0.13**	[0.70, 1.50]***
rsFC DMN	0.00	−0.01	[0.00, 0.00]	−0.00	−0.02	[−0.01, 0.00]	−0.00	−0.03	[−0.01, 0.00]
SES Hardship x rsFC DMN	−0.00	−0.01	[−0.00, 0.00]	−0.01	−0.02	[−0.04, 0.01]	−0.01	−0.01	[−0.05, 0.03]
ΔSleep Problems				**0.96**	**0.08**	[0.56, 1.35]***	0.30	0.03	[−0.50, 1.02]
R^2^		0.010***			0.140***			0.159	

b									
	ΔPoor Sleep Duration			ΔSuicidal Ideations (probit)			ΔSuicide Attempts (probit)		
Variables	b	β	95% CI[LB, UB]	b	β	95% CI[LB, UB]	b	β	95% CI[LB, UB]
**Unconditional Mediation**									
SES Hardship	**0.10**	**0.17**	[0.09, 0.12]***	**0.24**	**0.04**	[0.03, 0.44]*	**0.95**	**0.17**	[0.58, 1.34]***
T1 Anxious-Depressed	**0.03**	**0.04**	[0.01, 0.05]**	**1.32**	**0.16**	[1.07, 1.57]***	**1.21**	**0.14**	[0.84, 1.59]***
ΔPoor Sleep Duration				**0.35**	**0.04**	[0.01, 0.67]*	0.42	0.05	[−0.21, 1.00]
R^2^		0.031***			0.133***			0.157	
**Indirect Effects**									
SES Hardship → ΔPoor Sleep Duration → Suicide Risk									
			*Direct*	**0.24**	**0.04**	[0.03, 0.44]*	**0.95**	**0.17**	[0.58, 1.34]***
			*Indirect*	**0.04**	**0.01**	[0.00, 0.07]*	0.04	0.01	[−0.02, 0.10]
**Conditional Mediation**									
SES Hardship	**0.10**	**0.16**	[0.08, 0.12]***	**0.23**	**0.04**	[0.02, 0.43]*	**0.94**	**0.16**	[0.57, 1.32]***
T1 Anxious-Depressed	**0.03**	**0.04**	[0.01, 0.06]**	**1.32**	**0.16**	[1.07, 1.57]***	**1.21**	**0.14**	[0.84, 1.59]***
rsFC DMN	0.00	−0.00	[0.00, 0.00]	−0.00	−0.02	[−0.01, 0.00]	−0.00	−0.04	[−0.01, 0.00]
SES Hardship x rsFC DMN	−**0.00**	−**0.03**	**[**−**0.003**, −0.001]*	−0.01	−0.02	[−0.04, 0.01]	−0.01	−0.01	[−0.04, 0.03]
ΔPoor Sleep Duration				**0.34**	**0.04**	[0.00, 0.66]*	0.41	0.04	[−0.22, 0.99]
R^2^		0.034***			0.134***			0.159	
**Conditional Indirect Effects**									
SES Hardship x rsFC DMN → ΔPoor Sleep Duration → Suicide Risk									
			−*1 SD rsFC DMN*	**0.040**		[0.00, 0.08]*	0.048		[−0.03, 0.12]
			*Mean rsFC DMN*	**0.034**		[0.00, 0.07]*	0.041		[−0.02, 0.010]
			*+1 SD rsFC DMN*	**0.028**		[0.00, 0.06]*	0.041		[−0.02, 0.09]

Bootstrapping = 5000. Only primary paths of interest are shown to improve readability. Models controlled for youth age, biological sex, scanner motion, and baseline levels of sleep duration/problems, suicidal ideations, and suicide attempts. Complete estimates are available upon request. 95% confidence intervals are unstandardized.

*p < 0.05, **p <0.01, ***p <0.001.

We then tested the mediating effect of SES-H specifically on the sleep duration subscale, with SEM estimates for the sleep duration mediation model are presented in Table 2b. We found elevated levels of SES-H to be directly linked to an increased probability of youth reporting suicidal ideations (β = 0.04, 95% CI [0.01, 0.08], *p* < 0.05) and suicide attempts (β = 0.17, 95% CI [0.10, 0.22], *p* < 0.001) at the follow-up assessment. The influence of SES-H on the probability of suicidal ideations – but not suicide attempts – was mediated through worsening of youth sleep duration over the intermediary year (β = 0.17, 95% CI [0.14, 0.19], *p* < 0.001) over-and-above the effect of baseline youth anxiety/depressive symptoms (β = 0.04, 95% CI [0.01, 0.06], *p* < 0.01).

### Moderated mediation

**Fig. 2:**
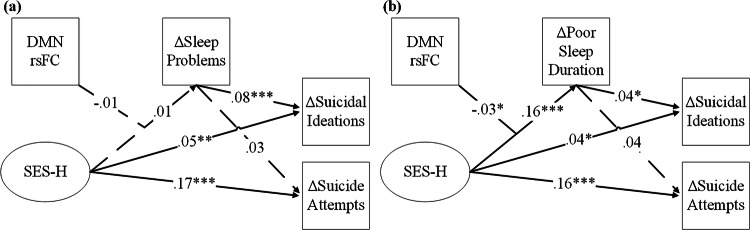
SEM Conditional Mediation Path Models. Note. **a** Suicide Risk via Sleep Problems. **b** Suicide Risk via Poor Sleep Duration. All path coefficients are standardized betas. ***p < 0.001, **p < 0.01, *p < 0.05.

**Fig. 3:**
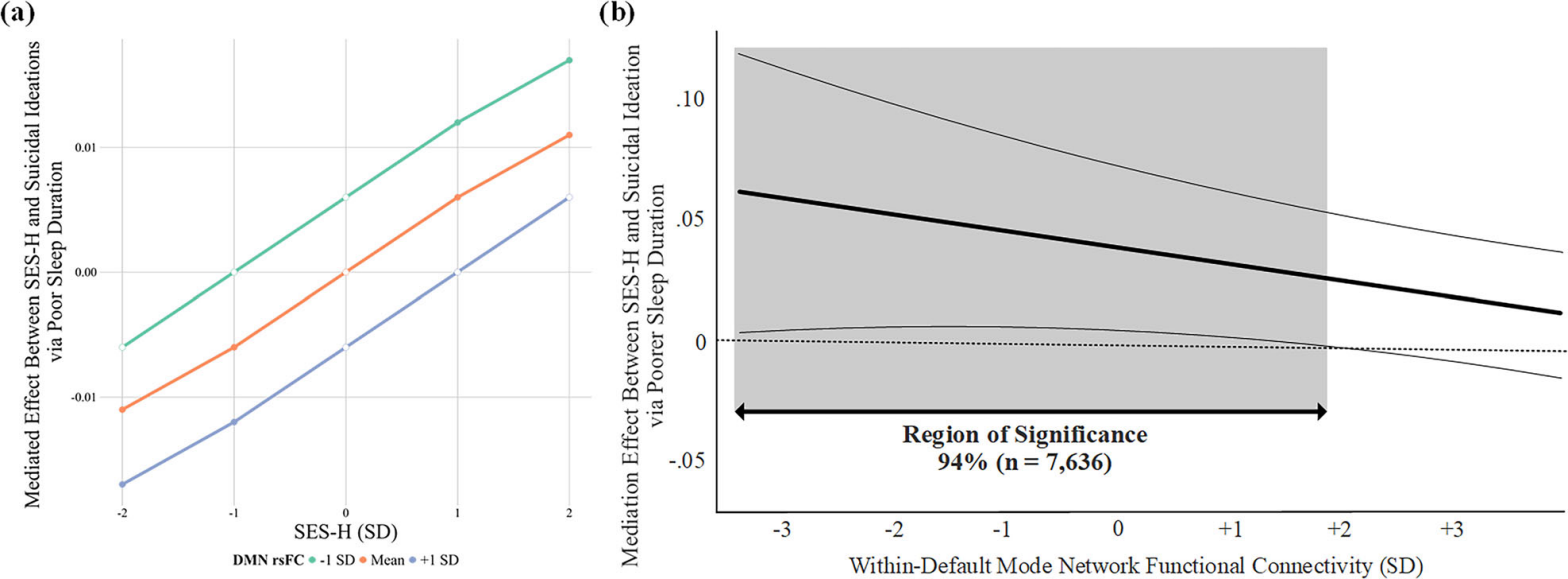
Conditional Indirect Effect of Harsh SES on Suicidal Ideations via Poor Sleep Duration. Note. **a** Pick-a-Point Plot of the Mediation Effect Conditioned on DMN rsFC. Filled dots indicate the indirect effect of the selected point is significant at *p* < 0.10. **b** Johnson-Neyman Plot of DMN rsFC Moderating the Indirect Effect of SES-H on Suicidal Ideations Through Poorer Sleep Duration.

Next, DMN rsFC was added into each mediation model as an a-path moderator to test if the effects of SES-H on later suicide risk through sleep health were conditional upon youth DMN coherence. As seen in Fig. 2a and b, we found the interactive effect of SES-H x rsFC DMN was a nonsignificant predictor of change in the sleep problems mediator (β = −0.01, 95% CI [−0.04, 0.01], *p* = 0.27), but it was associated with change in the sleep duration mediator (β = −0.03, 95% CI [−0.05, −0.01], *p* < 0.01). Specifically, as within-DMN rsFC coherence increased, the harmful effect that SES-H had on shortening youth sleep duration became weaker. The pick-a-point plot in Fig. 3a demonstrates how this interaction influenced the overall mediation pathway. That is, the increased risk that heightened SES-H has for youth to experience suicidal ideations through changes in sleep duration (i.e., the mediated effect) is attenuated as DMN rsFC coherence increases. The size of this effect was further probed using a Johnson-Neyman plot, revealing this protective conditional indirect effect to impact approximately 94% of the sample (n = 7636; Fig. 3b).

## Discussion

Past research indicates that SES-H is linked to adolescent risk for suicide. Limited prospective research has examined the mechanisms underlying this association, particularly among children and early adolescents. This study found that SES-H at age 10 forecasts increased suicidal ideation and attempts in early adolescence, in part due to reduced sleep duration. Notably, this developmental pathway is affected by DMN coherence. DMN coherence acts as a protective asset, buffering the influence of SES-H on youths’ sleep duration, which reduces the probability of experiencing suicidal ideations.

In our study, which utilized a large national sample, SES-H reported at age 10 predicted reduced sleep duration, and elevated suicidal ideation and attempts by age 11 or 12. Consistent with prior research, our findings highlight the role of SES-H in exacerbating sleep disturbances and increasing suicidal risk in young people [27, 49, 101–103]. These studies suggest that SES-H undermines sleep via influences on stress response systems and impairments in emotional regulation processes associated with suicidality [49, 103]. Mediational analyses support emerging bioregulatory perspectives on suicidal risk. Past research has focused on the role of affective distress and emotion regulation difficulties in linking SES-H to suicidality [104, 105]. We found that sleep health operates as a partial mechanism explaining SES-H effects. Sleep health reflects the physiological toll that adapting to harsh rearing environments exacts on children. Poor quality and diminished sleep undermine children’s development, compromising efforts to be involved in schooling and undermining emotion regulation and cognitive reserve needed to deal with maturation in a harsh environment [39]. Consequently, the interplay between SES-H, sleep health, and emotional dysregulation creates a pathway that elevates the risk of suicidal ideation and attempts among adolescents.

Study results revealed that sleep duration mediated the link between SES-H and suicidal risk; DMN coherence moderated the link between SES-H, sleep duration and suicidal ideation, but not sleep problems or suicide attempts. Youths with higher DMN coherence, relative to the examined sample, showed reduced probability of experiencing suicidal ideations in response to SES-H through relative improvements in their sleep duration. Study findings are consistent with past research documenting the protective role of DMN coherence in mitigating the association of early life stress on adolescent psychopathology [53], although this effect is inconsistently observed [106, 107]. The role of DMN coherence in moderating these associations offers new insights into the neurobiological mechanisms that might buffer the impacts of SES-H on sleep health and attendant suicidal ideation. Increased rsFC in brain regions linked specifically to self-referential thinking and emotional regulation may indicate a neurocognitive resilience mechanism [108], particularly for youth exposed to chronic stress and adversity [109]. Notably, the protective effect of within-DMN rsFC was less evident for suicide attempts, suggesting that different neurobiological processes or possibly DMN patterns may underlie different types of behavioral risk for suicide [108].

Our findings further underscore the importance of distinguishing between suicidal ideation and attempts as related yet distinct processes. Early identification of suicidal ideation in adolescents facing SES-H and sleep difficulties may present vital opportunities for intervention, potentially preventing escalation to attempts. Overall, findings in the present study highlight the importance of targeting sleep issues in interventions aimed at reducing suicidal risk among youth facing economic adversity.

### Clinical implications

Study findings underscore the need for sleep assessment in pediatric and adolescent populations, particularly among those living in under-resourced environments, where sleep disruptions may contribute to heightened suicide. Sleep problems constitute an achievable focus for clinicians, families, and communities seeking to improve youth mental health and reduce suicide risk. Screening for suicidal ideation should be prioritized in individuals presenting with poor sleep quality or insufficient sleep, regardless of clinical setting. The documented protective effect of DMN coherence is intriguing but requires replication and further research on interventions that influence global processing networks. Given that DMN coherence typically increases across adolescence, identifying strategies that promote normative maturation in connectivity early in development may play a crucial role in mitigating suicide risk, especially in low-resource settings.

### Limitations and strengths

This study has several limitations that should be acknowledged. First, although the longitudinal design allows for examining temporal associations between SES-H, sleep duration, and suicidal risk, causality cannot be definitively established due to the non-experimental nature of the study. Relatedly, our results underscore the salient effects of SES-H, DMN rsFC and sleep duration in predicting the probability of later suicidal ideations. Yet, the nonsignificant effects for suicide attempts may be attributable to a lack of statistical power given the relatively small number of endorsements of suicide attempts among youth at the follow-up assessment (n = 137, 1.7%), raising concerns about possible low power to detect these effects. Additionally, consistent with ABCD recommendations, we excluded children with missing neuroimaging data or poor-quality scans. However, this criterion may have introduced demographic biases, including differential exclusion by race, and may have led to missing identification of differences in age, sex, race/ethnicity, and SES [110]. Future studies are needed that focus on populations that are not proportionally represented herein and consider sex-stratified models, given potential neurodevelopmental differences by biological sex.

Other limitations arise from how study variables were operationalized. Specifically, the reliance on parent-reported sleep, which, while practical and commonly used in large studies, limits precision compared to actigraphy or youth self-reports and may overlook subtle disturbances like insomnia or late-night device use [111]. This can introduce error or weaken associations with brain and behavioral outcomes. At the time, parent reports were the only feasible option, but future work should incorporate actigraphy or youth self-reports for greater accuracy. Last, the latent variable approach employed to characterize SES-H enabled the qualities of a broader rearing context to be modeled; however, it did not include higher-level ecological or neighborhood-level indicators of hardship. Future research can employ other rigorous measurement methods (e.g., multilevel frameworks, neighborhood-focused hypotheses, or exposome approaches) to further examine the role of SES-H on the development of adolescent STB.

## Supplementary information

Figure_S1

Table_S1

Table_S2
