# Supplementary figures and images for: Neurobehavioral pathways linking socioeconomic status hardship to suicide risk versus resilience in young adolescents: the roles of sleep health and default mode network connectivity
Source: Transl Psychiatry. 2025 Nov 24;15:497. doi: 10.1038/s41398-025-03710-y (PMC12644833; doi:10.1038/s41398-025-03710-y)

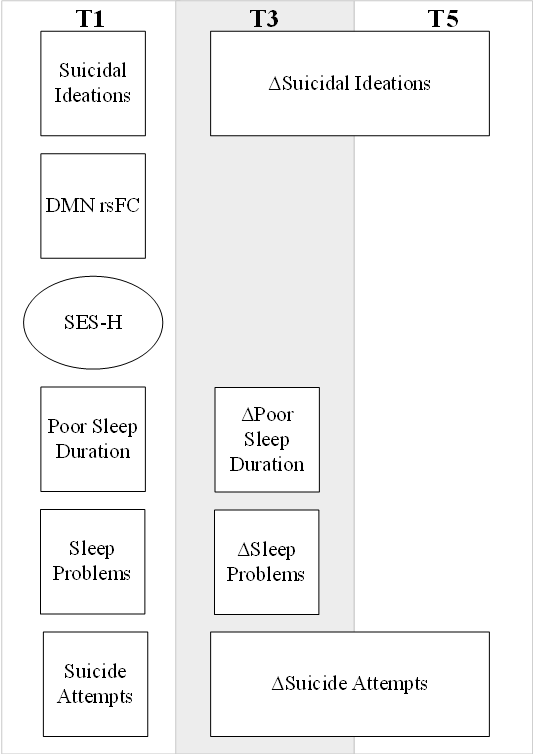

Supplement: Supplementary file 1 — Figure_S1 [file 41398_2025_3710_MOESM1_ESM.png]
